# Survival after venoarterial extracorporeal membrane oxygenation for acute high-risk Pulmonary Embolism: A meta-analysis of comparative studies

**DOI:** 10.12669/pjms.41.1.10568

**Published:** 2025-01

**Authors:** Xiaoying Yang, Min Xiang, Lifang Wu, Meng He, Hongyan Zhu

**Affiliations:** 1 Xiaoying Yang, Pulmonary and Critical Care Medicine Dept., Shaoxing People’s Hospital, Shaoxing, Zhejiang Province312000, P.R. China; 2 Min Xiang, Pulmonary and Critical Care Medicine Dept., Shaoxing People’s Hospital, Shaoxing, Zhejiang Province312000, P.R. China; 3 Lifang Wu, Department of Infectious Diseases, Shaoxing People’s Hospital, Shaoxing, Zhejiang Province312000, P.R. China; 4 Meng He, Pulmonary and Critical Care Medicine Dept., Shaoxing People’s Hospital, Shaoxing, Zhejiang Province312000, P.R. China; 5 Hongyan Zhu, Hospital Infection Management Division, Shaoxing People’s Hospital, Shaoxing, Zhejiang Province312000, P.R. China

**Keywords:** Thromboembolism, Extracorporeal Membrane Oxygenation, Mortality, Critical care

## Abstract

**Objective:**

The survival benefit of venoarterial Extracorporeal Membrane Oxygenation (ECMO) for the management of acute high-risk pulmonary embolism (PE) remains unclear. This meta-analysis combines data from comparative studies to assess the risk of mortality after ECMO vs standard care in the management of acute high-risk PE.

**Methods:**

Databases of PubMed, CENTRAL, Scopus, Web of Science, and Embase were searched from 01^st^ January 2000 to 24^th^ March 2023 for comparative studies with at least 10 patients/group comparing ECMO vs standard treatment. PRISMA guidelines were followed for this review. We extracted mortality data and combined it to obtain the outcome as risk ratios (RR) with 95% confidence intervals (CI) in a random-effects model.

**Results:**

Ten studies were included. Meta-analysis showed that the risk of early mortality was not significantly different between ECMO and non-ECMO groups (RR: 0.97 95% CI: 0.78, 1.19 I^2^=73%). No change in significance was noted on subgroup analysis based on study location, sample size, cardiac arrest, systemic thrombolysis, and surgical embolectomy.

**Conclusion:**

Limited evidence derived from mostly retrospective studies riddled with selection bias suggests that ECMO may not offer additional survival benefits in high-risk PE.

## INTRODUCTION

Acute pulmonary embolism (PE) remains a widely prevalent thromboembolic disorder.^1^ Despite the availability of improved diagnostic procedures and modern therapeutic modalities the morbidity and mortality of PE remain high.^2^ The clinical presentation of PE ranges widely from high risk or massive PE to low risk of sub-massive/non-massive PE depending upon the degree of right ventricular dysfunction and hemodynamic instability.^3^ Concomitantly, the mortality rates also show considerable variation from around one percent^3^ for the least severe cases to 71.4% for high-risk PE.^4^ High-risk PE accounts for five percent of all cases of PE.^3^

In such situations, early systemic thrombolysis or catheter-directed therapy (CDT) or surgical thrombectomy with conventional anticoagulation therapy has been shown to reduce mortality rates.^5^ Indeed, systemic thrombolysis has been the first line of therapy for high-risk PE with a meta-analysis showing up to a 61% reduction in mortality.^6^ Nevertheless, not all patients with PE are candidates for thrombolysis and it remains underused even in patients with hemodynamic instability.^7^ Also, contraindications and failure with thrombolysis and high-risk bleeding with thrombolytic treatment are significant limitations.^8^

Indeed, where thrombolytic therapy has failed or is contraindicated venoarterial extracorporeal membrane oxygenation (ECMO) can serve as a bridging therapy for CDT or surgical thrombectomy. By providing complete mechanical circulatory support and oxygenation of circulating blood, ECMO can provide hemodynamic stability to PE patients presenting with shock or cardiac arrest.^9^,^10^ However, evidence for ECMO in improving outcomes is mostly based on case reports and case series. It is still unclear if ECMO provides survival benefits compared to standard care for PE patients. In the past two meta-analysis studies on comparative cohorts have attempted to examine the impact of ECMO on survival but included limited studies, many of them with very small sample sizes.^11^,^12^ To overcome the limitations and provide updated evidence, we conducted the present study to compare mortality rates with and without ECMO in high-risk PE patients.

## METHODS

PROSPERO registration of the study was done (CRD42023409705) (https://www.crd.york.ac.uk/prospero). The authors made sure that the PRISMA statement reporting guidelines were observed.^13^ An online database search was undertaken on PubMed, CENTRAL, Scopus, Web of Science, and Embase. All English language studies conducted on humans and published from 01^st^ January 2000 to 24^th^ March 2023 were considered for inclusion.

### Inclusion criteria:

- Studies on acute massive or high-risk PE patients defined by any standard criteria were included (*Population*).
- The intervention was the use of ECMO.
- The comparison was standard care without ECMO.
- The outcome of interest was the mortality rate.

### Exclusion criteria:

- Studies with less than 10 patients in each group.
- Studies not reporting relevant data.
- Studies not comparing ECMO with control.
- Studies with duplicate/overlapping data.
- Review articles, case reports, case series, and editorials.

A mix of free-text and MeSH keywords with Boolean operators (AND/OR) were utilized for the literature search. The search terms included “pulmonary embolism”, “high-risk”, “massive”, “extracorporeal”, “survival”, and “mortality”. PubMed search strategy is presented in detail in Supplementary Table-I.

**Table-I:** Details of included studies.

Study	Location	Type	Included patients	Groups	Sample size	Age	Male gender (%)	Shock	Cardiac arrest	ST (%)	SE (%)	Bleeding (%)	Infection (%)	NOS
Bougouin 2017^15^	France	P	PE -related sudden cardiac arrest	ECMO	12	60.8*	46*	NR	100*	57*	0*	NR	NR	6
				Control	70									
Funakoshi 2018^18^	Japan	R	AHA defined massive PE as PE with sustained hypotension requiring inotropic support	ECMO	112	58.5	37.5	NR	43 18	74*	26*	NR	NR	6
				Control	249	65.9	37.8							
Meneveau 2018^17^	France	R	ESC defined suspected or confirmed high risk PE	ECMO	52	47.6	52	25	75	32.7	32.7	38.5	NR	6
				Control	128	64	54	45.3	35.1	53	6.3	6.3		
Minakawa 2018^16^	Japan	R	Acute massive PE undergoing SE	ECMO	94	62.1*	47.6*	50.1*	NR	NR	100*	NR	NR	6
				Control	261									
Kjaergaard 2019^20^	Denmark	R	Massive PE	ECMO	22	NR	NR	23	100	55*	23*	14*	NR	6
				Control	16			88	0					
Madingers 2019^19^	Netherlands	R	Massive PE	ECMO	22	40	41	NR	100	86	NR	68	24	6
				Control	46	57	50.8		100	59		9	2	
Goldberg 2021^23^	USA	R	AHA defined massive PE	ECMO	27	55.7	37	NR	NR	29.6	0	0	0	6
				Control	32	55.4	62.5			6.3	100	3.1	0	
Panholzer 2022^22^	Germany	R	Acute PE undergoing SE	ECMO Control	15 88	58.4*	58.2	NR	NR	12.6*	100*	8 5	NR	6
Tsai 2022^21^	Taiwan	R	Massive PE	ECMO	25	52	68	NR	60	36	0*	52	NR	6
				Control	15	73	33.3		73.3	20		45.5		
Nasser 2023^24^	USA	R	High-risk PE	ECMO	780	NR	NR	NR	NR	NR	NR	NR	NR	8
				Control	780									

AHA, American Heart Association; ESC, European Society of Cardiology; SE, Surgical embolectomy; ST, systemic thrombolysis; ECMO, Extracorporeal Membrane Oxygenation; P, prospective; R, retrospective; PE, pulmonary embolism; NR, not reported; NOS, Newcastle Ottawa scale.

* overall population data.

We collected all the search results in a reference manager software. All duplicate studies were then removed electronically. The remaining records were carefully inquired about based on the eligibility criteria by two reviewers separately. This was done first at the title/abstract level and then at the full-text level. Articles completing all eligibility criteria were included. Any disagreements were solved by consensus. The references list of eligible articles was hand searched for additional articles.

### Data management and study quality:

Data on the author’s last name, year of publication, study location, study type, included patients, sample size, age, gender, shock, cardiac arrest, systemic thrombolysis, surgical embolectomy, bleeding, infection rates, and outcome data were extracted by two reviewers independent of each other.

Two authors judged the study’s quality based on Newcastle Ottawa Scale (NOS).^14^ The NOS has three domains: representativeness of the study cohort, comparability, and measurement of outcomes. Points are given based on the preformatted questions.

### Statistical analysis:

Statistical analysis was done using “Review Manager” (RevMan, version 5.3; Nordic Cochrane Centre (Cochrane Collaboration), Copenhagen, Denmark; 2014). We extracted mortality data and combined it to obtain the outcome as risk ratios (RR) with 95% confidence intervals (CI) in a random-effects model. Publication bias was examined using funnel plots. The I^2^ statistic was the tool to determine inter-study heterogeneity. I^2^ <50% meant low and >50% meant substantial heterogeneity. A leave-one-out analysis was performed to check for any change in the results on the exclusion of any study. Subgroup analysis was done based on study location, sample size, cardiac arrest, systemic thrombolysis, and surgical embolectomy. P-value <0.05 was considered statistically significant.

## RESULTS

**Fig.1:**
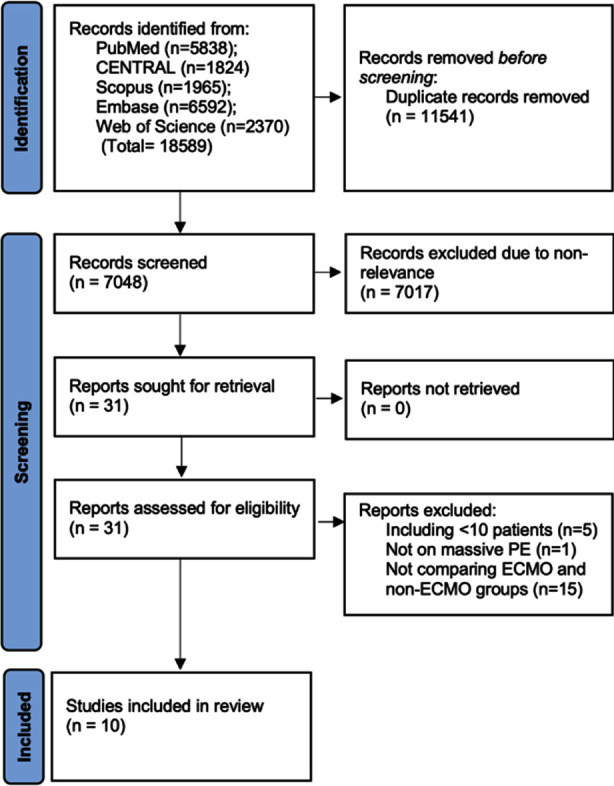
Study flowchart.

Records obtained during the literature search are shown in Fig.1. At first, 18589 studies were retrieved. Duplicates amongst those were removed leaving 7048 results. The reviewers examined these articles for primary eligibility and 7017 were excluded due to non-relevance. The 31 studies which were selected for full-text analysis underwent detailed examination and 10 were found to be appropriate based on the inclusion criteria^15^-^24^. The remaining 21 studies were excluded for reasons mentioned in Fig.1.

**Fig.2:**
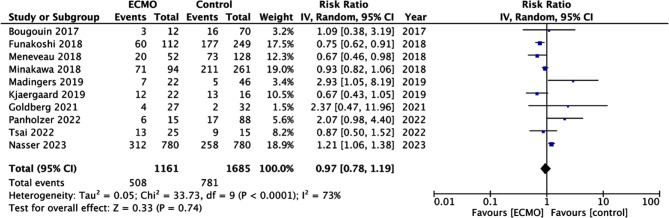
Meta-analysis of mortality between ECMO and non-ECMO groups.

The majority of studies were conducted in Western countries with only three^16^,^18^,^21^ on Asian populations (Table-I). The total sample size of the studies was 2846. Only one study by Nasser et al^24^ used propensity score matching to match the ECMO and control groups. In three studies^15^,^19^,^20^, 100% of patients receiving ECMO were in cardiac arrest. The use of systemic thrombolysis varied from 6.3 to 86%. In two studies^16^,^22^, all patients underwent surgical embolectomy, while in another two^15^,^21^ none of the patients underwent the surgical procedure. In the study of Goldberg et al,^23^ only control group patients underwent surgical embolectomy. All studies reported data on in-hospital or early mortality. The quality of studies was not high as majority received a NOS score of six. Meta-analysis showed that the risk of early mortality was not significantly different between ECMO and non-ECMO groups (RR: 0.97 95% CI: 0.78, 1.19 I^2^=73%) (Fig.2). The forest plot did not demonstrate any publication bias (Supplementary Fig.1). Sensitivity analysis did not change the results (Table-II).

**Table-II:** Results of sensitivity analysis.

Excluded study	Risk ratio (95% Confidence intervals)
Bougouin 2017^15^	0.96 (0.78, 1.19) I^2^=76%
Funakoshi 2018^18^	1.02 (0.81, 1.27) I^2^=68%
Meneveau 2018^17^	1.01 (0.81, 1.26) I^2^=73%
Minakawa 2018^16^	1.00 (0.76, 1.33) I^2^=76%
Kjaergaard 2019^20^	1.01 (0.81, 1.25) I^2^=74%
Madingers 2019^19^	0.93 (0.76, 1.13) I^2^=73%
Goldberg 2021^23^	0.96 (0.77, 1.17) I^2^=75%
Panholzer 2022^22^	0.92 (0.75, 1.13) I^2^=73%
Tsai 2022^21^	0.98 (0.78, 1.22) I^2^=76%
Nasser 2023^24^	0.90 (0.73, 1.12) I^2^=57%

The outcomes of various subgroup analyses are demonstrated in Supplementary Table-II. The results remained non-significant based on study location (Western or Asian), sample size (>50 or <50 patients in ECMO group), presence of cardiac arrest (all patients or mixed), systemic thrombolysis (>50% or <50% of the cohort), and surgical embolectomy (all patients or mixed).

## DISCUSSION

High-risk and intermediate-risk PE often requires interventional therapies like systemic thrombolysis or CDT, or surgical thrombectomy for management.^5^ Further, mortality with high-risk PE remains considerably high.^3^ In these conditions, ECMO can provide hemodynamic support by completely bypassing the pulmonary circulation and enhancing tissue oxygenation.^9^,^10^ The recent American Heart Association scientific statement states that high-risk PE patients who require interventional strategies may be supported by ECMO while these therapies are administered.^25^ The European Society of Cardiology has given a Class IIB recommendation for ECMO use amongst those with cardiac arrest or shock requiring CDT or surgical thrombectomy.^26^

Several authors have reported their experience with the use of ECMO for high-risk PE. Baldetti et al^27^ have compiled data from 21 single-arm studies to show that in the real world, ECMO was used for high-risk PE with cardiac arrest in 62.3% of cases while immediate ECMO was provided in 61.9% of cases. Overall, the mortality rate was 41.1%. Nevertheless, pooled analysis of single-arm studies does not provide clarity vis-à-vis standard care of high-risk PE. Karami et al^12^ conducted a meta-analysis of nine studies (353 patients) to show no difference in the risk of early mortality between high-risk PE patients receiving ECMO or standard care. However, three of their included studies had a very small sample size (<10 patients). Kaso et al^11^ conducted a meta-analysis of 11 comparative studies to show no impact of ECMO on early mortality rates.

Nevertheless, their review had significant limitations. Firstly, four studies had an extremely small sample size (ECMO patients ranging from two to seven). Secondly, two^10^,^28^ of the included studies were single-arm studies. Given such major shortcomings, the overall reliability of evidence is considerably reduced. Research has shown that very small sample size studies are a major threat to the validity of meta-analyses owing to their large treatment effects and publication bias.^29^ Thus, in this review, we not only excluded very small sample size studies but also conducted an updated literature search to include 10 studies with a total of 1161 patients thereby providing the most optimal evidence. Consistent with the outcomes of prior reviews, we noted no difference in the risk of early mortality in high-risk PE patients receiving ECMO vs standard care. The overall mortality rate in the ECMO group was 43.7% which concurs with the meta-analysis of single-arm studies of Baldetti et al.^27^

The lack of difference in mortality with and without ECMO should not be interpreted as the inefficacy of ECMO in improving the survival of high-risk PE patients. It must be remembered that the current as well as previous meta-analyses studies^11^,^12^ are based mostly on retrospective data which has inherent selection bias. Baseline variations in patient characteristics and physician’s judgment could have a major role in the selection of patients for ECMO and therefore outcomes. It has been suggested that ECMO is largely used in cases with cardiac arrest as a salvage therapy.^11^

In our review, in three of the included studies, the incidence of cardiac arrest was higher in the ECMO compared to the control group while many others failed to report such important data. Contrastingly, a study by George et al^30^ has suggested that ECMO may not benefit those with cardiac arrest and blood lactate > 6 mmol/L. Similarly, Tsai et al^21^ have shown no survival benefit of ECMO in patients with cardiac arrest but excellent results amongst those without cardiac arrest. Their study also showed that timing of ECMO is an important factor with maximum survival benefit seen with early ECMO. Another aspect to consider is the reperfusion strategy used after ECMO which can affect patient survival. Chopard et al^31^ have recently demonstrated that mechanical embolectomy after ECMO results in significantly better outcomes as compared to other reperfusion strategies or ECMO as a stand-alone therapy. In our review, many of the studies failed to report the reperfusion strategy. The variations in patient populations, baseline comorbidities, and treatment protocols could have further added to the heterogeneity. Nevertheless, an attempt was made to explore its cause by conducting multiple subgroup analyses all of which failed to change the significance of the results or reduce the heterogeneity.

We failed to conduct a meta-analysis of ECMO complications due to a lack of adequate data from the included cohorts. Indeed, a major cause of mortality after ECMO is due to its associated complications like bleeding, thrombosis, or access-site concerns. Bleeding can occur in up to 70% of cases receiving ECMO and is proportionate to higher death rates.^32^ Secondly, bypassing of the pulmonary circuit in ECMO patients who have minimal residual cardiac function can result in thrombosis. Also, ECMO can lead to intravascular coagulation problems due to increased consumption of platelets and coagulation factors by shear stress.^33^ Furthermore, major illness and systemic lysis can induce changes in clotting time and activated partial thromboplastin time which makes monitoring of unfractionated heparin difficult thereby increasing bleeding risk with ECMO.^24^

### Limitations:

Given the retrospective nature of the data, results must be interpreted with caution. Furthermore, only one study^24^ used propensity-score matching to take into account baseline differences. We could also assess only crude mortality rates as multivariate-adjusted data was also unavailable. Secondly, the number of studies in the review was not high. Also, only early mortality rates could be assessed as data for late mortality and other complications were not uniformly reported. Thirdly, the high interstudy heterogeneity is also a cause of concern.

Nevertheless, the strength of the study is that it is the most updated systematic review providing comparative evidence for ECMO vs non-ECMO based management of high-risk PE patients. Compared to the prior meta-analysis studies,^11^,^12^ we excluded very small sample size studies and conducted an updated literature search to include additional large sample size studies to generate better evidence. We also tested the reliability of the outcomes by conducting sensitivity and subgroup analyses. We believe that our study provides important preliminary evidence on the efficacy of ECMO in high-risk PE patients. While it suggests that use of ECMO may not improve survival, the outcomes should not be interpreted with caution, given the retrospective nature of the data. We suggest that clinicians should continue using ECMO based on individual patient requirements till further high-quality evidence is available. The present study should provide impetus to researchers to further investigate the role of ECMO in high-risk PE. Future studies using propensity-score matching data should be conducted to assess if an expensive and intensive treatment like ECMO leads to survival benefits in high-risk PE.

## CONCLUSIONS

Limited evidence derived from mostly retrospective studies riddled with selection bias suggests that ECMO may not offer additional survival benefits in high-risk PE. Future studies with large sample sizes taking into account confounding factors are needed to generate quality evidence.

***Funding:*** 2023 Zhejiang Province Traditional Chinese Medicine Science and Technology Plan Project (2023ZL183), 2022 Shaoxing City Health Science and Technology Plan Project (2022KY006).

### Authors’ contributions:

**XY:** Conceived and designed the study.

**MX**, **LW**, **MH** and **HZ:** Literature search, Collected the data and performed the analysis.

**XY:** Literature search, writing of the manuscript and is responsible for the integrity of the study.

All authors have read and approved the final manuscript.

***Note: Information Regarding Supplementary Tables & Figures can be Obtained from the correspondence author*.**
